# Our Advertising Department

**Published:** 1885-01-20

**Authors:** 


### OUR ADVERTISING DEPARTMENT

Will be found interesting. Many regard the advertisements in a journal as among the most desirable items of information. As the amount of reading matter in our journal remains uniformly the same, the advertising costs the subscriber nothing, while they serve largely to assist the editors in maintaining the heavy expenses incident to journalism.

